# Role of Inflammation in Human Fatigue: Relevance of Multidimensional Assessments and Potential Neuronal Mechanisms

**DOI:** 10.3389/fimmu.2017.00021

**Published:** 2017-01-20

**Authors:** Bianka Karshikoff, Tina Sundelin, Julie Lasselin

**Affiliations:** ^1^Division for Psychology, Department of Clinical Neuroscience, Karolinska Institutet, Solna, Sweden; ^2^Stress Research Institute, Stockholm University, Stockholm, Sweden; ^3^Department of Psychology, Stockholm University, Stockholm, Sweden; ^4^Institute of Medical Psychology and Behavioral Immunobiology, University Hospital Essen, Essen, Germany

**Keywords:** central fatigue, inflammation, immune system, multidimensional assessments, motivation, ventral striatum, anterior cingulate cortex, insula

## Abstract

Fatigue is a highly disabling symptom in various medical conditions. While inflammation has been suggested as a potential contributor to the development of fatigue, underlying mechanisms remain poorly understood. In this review, we propose that a better assessment of central fatigue, taking into account its multidimensional features, could help elucidate the role and mechanisms of inflammation in fatigue development. A description of the features of central fatigue is provided, and the current evidence describing the association between inflammation and fatigue in various medical conditions is reviewed. Additionally, the effect of inflammation on specific neuronal processes that may be involved in distinct fatigue dimensions is described. We suggest that the multidimensional aspects of fatigue should be assessed in future studies of inflammation-induced fatigue and that this would benefit the development of effective therapeutic interventions.

## Introduction

Fatigue is a highly disabling symptom that is common in various medical and psychiatric conditions (1–4). In some cases, the origin of fatigue can be explained by alterations in muscle metabolism or the cardiovascular system, but for most clinical populations, such as cancer survivors and patients suffering from multiple sclerosis (MS) or chronic fatigue syndrome/myalgic encephalomyelitis (CFS/ME), fatigue pathophysiology remains hard to establish. In these conditions, inflammation has been hypothesized as a possible contributor (5, 6), based on an extensive literature showing the capacity of inflammatory factors to act on the central nervous system (CNS) and induce behavioral changes, including fatigue (7–9). Furthermore, alterations in inflammatory processes are found in patients suffering from medical conditions also characterized by high rates of fatigue, such as cancer survivors and patients with MS or diabetes (6). However, despite reported associations between fatigue and circulating levels of inflammatory markers in populations of patients suffering from these diseases (10, 11), the specific role and biological mechanisms of inflammation in the development of fatigue symptoms remain elusive. One of the reasons may be that fatigue is rarely assessed from a multidimensional perspective.

## Fatigue: A Multidimensional Perspective

### Definitions of Fatigue

The multidimensional aspect of fatigue has been addressed in previous literature. One distinction relates to *peripheral* and *central* fatigue (12), with peripheral fatigue described as “the inability to sustain a specified force output or work rate during exercise,” and central fatigue as “the failure to initiate and/or sustain attentional tasks and physical activities requiring self-motivation” (13). This distinction thus lies mainly in the origin of the feeling, with peripheral fatigue developing from peripheral physiological and neuronal systems (e.g., neuromuscular transmission, muscular metabolism, or the cardiovascular system), whereas central fatigue results from changes in the CNS. Central fatigue is further comprised of several dimensions, namely *physical fatigue, mental/cognitive fatigue*, and *motivational changes*. Physical fatigue is characterized by a difficulty in performing physical activities, while mental/cognitive fatigue is described as difficulties concentrating and carrying out cognitive tasks (14). These distinctions reflect the behavioral outputs of central fatigue. For these behaviors, motivational changes appear to be central. Motivational inputs, such as expected rewards and benefits, modulate the effort exerted by the individual in any given situation (15). Hence, fatigue has been suggested to arise when the balance between the energy costs and the expected reward of an action is disrupted (16). Consequently, central fatigue may depend on flawed integrations of motivational inputs and/or energy expenditure (13, 15).

Another important aspect to take into account regarding central fatigue is the distinction between *physiological* and *pathological* fatigue. Biologically, fatigue is first and foremost an adaptive physiological process. It is the reduction of effort, resulting from perceived exertion (appraised by motor and sensory inputs) and motivational factors (15). Fatigue is a signal to rest, and it encourages energy preservation to prevent injuries, which may be beneficial after intense work or sleep loss, or when the bodily resources need to be redirected toward fighting pathogens during an infection (17). Fatigue also helps focus on more energy-efficient actions (16). As such, healthy, normal physiological processes of fatigue are denoted *physiological fatigue* in this review, as opposed to *pathological fatigue*, which is a state where the adaptive function has been lost. Although central fatigue is primarily a feeling, and usually assessed through subjective measurements (e.g., self-report questionnaires), it can also be measured objectively, using physical, cognitive, or motivational tasks.

**Figure 1:**
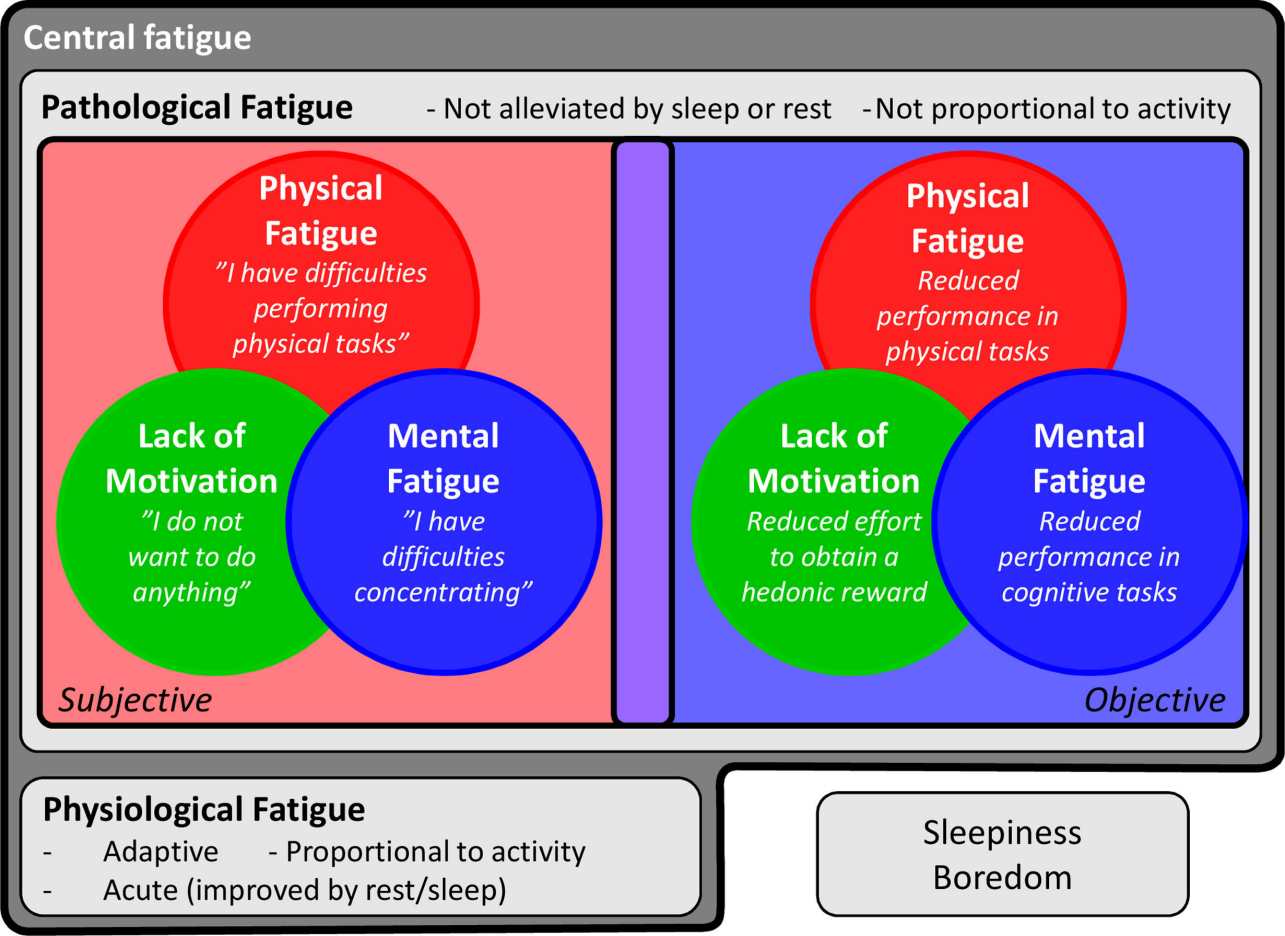
**Features of central fatigue**. Central fatigue is a complex symptom including several dimensions and concepts. It can be divided into *physiological* fatigue, a signal to rest and encourage energy preservation to prevent injuries, and *pathological* fatigue, when the adaptive function has been lost. Central fatigue is also comprised of several dimensions, namely *physical fatigue, mental fatigue*, and *lack of motivation*. These dimensions can be assessed in subjective or objective ways. Finally, the study of fatigue is further complicated by the difficulty in separating fatigue from close but distinct concepts, such as sleepiness and boredom.

Taken together, central fatigue appears to be not just “fatigue,” but a complex symptom that comprises several dimensions and concepts (Figure 1). In this review, we will focus on the effects of inflammation on central fatigue and illustrate the importance of multidimensional assessments in understanding the pathophysiology of inflammation-induced central fatigue.

### Issues to Consider When Studying Fatigue

Fatigue is a highly subjective experience that every human being experiences at some point. This intuitive everyday understanding of fatigue may complicate formal assessments. A subjective distinction between normal (physiological) but pronounced fatigue *versus* pathological fatigue may be difficult to describe and, as such, the nature and intensity of pathological fatigue may be difficult to understand for relatives and caregivers (18). Furthermore, in everyday speech, fatigue is often used interchangeably with tiredness, which in turn is used as a synonym for sleepiness, i.e., sleep propensity (19, 20). Although fatigue and sleepiness are generally considered different concepts in both research and clinical practice, some assessment scales use sleepiness as a dimension of fatigue (21), and some tasks that induce mental fatigue also cause sleepiness (22, 23). This relationship is further complicated by the concept of tiredness, which may be considered equal to fatigue or as a lesser version thereof (24). In addition, other feelings can also be interpreted as—and overlap with—fatigue, such as boredom (25). Evidently, there is a need for a clear characterization of fatigue, both physiological and pathological. The literature on diseases in which fatigue is one of the main causes of suffering for the patients, such as cancer, may help in this regard. The interdisciplinary workgroup Assessing the Symptoms of Cancer using Patient-Reported Outcomes highlights several characteristics of cancer-related fatigue (26), some of them appearing critical for distinguishing pathological from physiological fatigue. For example, as opposed to physiological fatigue, pathological fatigue is not alleviated by sleep or rest (18) and is not proportional to the degree of activity (27).

Beyond the distinction between physiological and pathological fatigue, the assessment of fatigue should be performed keeping in mind the several dimensions and conflicting or overlapping concepts, as discussed above (Figure 1). These dimensions may in fact involve distinct neuronal systems (see Part 3 of the current review). What is called “fatigue” may thus be driven by different underlying mechanisms from one patient to another and from one condition to another. While some self-assessment scales encompass several kinds of fatigue [e.g., the multidimensional fatigue inventory (MFI) (14)], others rely on single or non-specific aspects and, although having clinical relevance, may prevent the understanding of the pathophysiological processes. In addition, while the use of long or intense physical or cognitive tasks assesses fatigue in an objective way, these objective measures do not always correlate with subjective measures, indicating that they may actually assess distinct components (28) and may not be ecologically valid.

Given the large clinical overlap between pain and fatigue (29), the conceptualization of fatigue could be inspired by that of pain. In pain research, both central and peripheral biological components have been identified (30), as well as a fairly well-described neuronal network (31). There are clear mechanistic differences between acute and chronic pain (32), and, depending on the diagnosis, peripheral and central dysfunctions are involved to different degrees (33). However, some components are common for all pain diagnoses (34), and low-grade inflammation has recently been added to this list (35, 36). Following this rationale of pain research, we suggest that there are identifiable biological mechanisms that drive fatigue and that these include peripheral and central components, as well as identifiable neuronal networks. Moreover, the mechanisms may change if the fatigue becomes chronic and may vary for different conditions. Specifically, we argue that inflammation affects some of the biological systems underlying fatigue and that inflammation may therefore be one of the major driving forces for fatigue.

## Inflammation and Fatigue

### The Activated Immune System Induces Fatigue

During activation of the immune system, immune cells produce pro- and anti-inflammatory cytokines, which are signaling molecules that coordinate the fight against the pathogen. A less-known feature of cytokines is their capacity to act on the CNS, inducing behavioral alterations, including the development of fatigue [for reviews, see Ref. (7, 8)]. The cytokine signal reaches the brain *via* several immune-to-brain pathways, e.g., by a neuronal pathway *via* the vagus nerve or by a humoral pathway *via* brain locations with a weaker blood–brain barrier (37–39). Cytokines in the brain then induce modifications in neurotransmitter and neuroendocrine systems (Box 1), along with modifications in brain functions, which lead to behavioral changes.

Box 1 **Cytokine effects in the brain.**

During the activation of the immune system, immune cells produce cytokines that coordinate the immune response. In addition to their peripheral actions, cytokines are able to signal to the CNS. Several such immune-to-brain communication pathways have been described, including a *neuronal* and a *humoral* pathway. The former refers to the fact that cytokines can activate the vagus nerve at the periphery, which then modulates functions of the brain targets of vagal afferents (38). Cytokines can also activate brain immune cells (microglia) that are located alongside brain vessels where the blood–brain barrier is weaker (e.g., in the circumventricular organs) (39). These cells then produce cytokines locally. The cytokine signal propagates in the brain *via* diffusion, microglial activation, and neuronal projections (37).

A crucial mechanism by which cytokines modulate neuronal functions is through modifications of monoaminergic neurotransmission, specifically by activating enzymes interfering with dopamine and serotonin biosynthesis. One of these enzymes is the GTP-cyclohydrolase 1 (GTP-CH1), which is involved in the production of neopterin. The production of neopterin happens at the expense of the production of tetrahydrobiopterin (BH4), which is an essential cofactor for the biosynthesis of dopamine and serotonin (40, 41). In addition, cytokines activate the indoleamine 2,3-dioxygenase (IDO), the rate-limiting enzyme degrading tryptophan along the kynurenine pathway (7, 42). The degradation of tryptophan reduces its availability for serotonin biosynthesis. By activating GTP-CH1 and IDO, cytokines thus reduce the synthesis of dopamine and serotonin. Cytokines also modulate dopamine- and serotonin-transporter activity, reducing their synaptic availability (43, 44). In addition to neurotransmitter systems, cytokines modulate neuroendocrine systems, such as the hypothalamic–pituitary–adrenal axis, activating the release of corticotropin-releasing hormone, adrenocorticotropic hormone, and cortisol (45, 46).

Although cytokines induce a large array of behavioral changes, including changes in mood and cognitive functions, fatigue is, interestingly, one of the first and most common symptoms associated with an activated immune system (47). This has been demonstrated in patients suffering from cancer or hepatitis C, who undergo immunotherapy with the pro-inflammatory cytokine interferon-α, which activates the immune system and has neuropsychiatric side effects (48–50). Among these, fatigue develops very rapidly after instauration of the treatment in a large proportion (up to 80%) of patients, while other behavioral alterations, such as depressed mood and cognitive dysfunction, appear later and only in a subpopulation (30–60%) of patients (49, 51). This suggests that fatigue is very sensitive to the effects of cytokines, and underscores the biological connection between fatigue and inflammation.

### Clinical Aspects of Inflammation and Fatigue

There is increasing evidence supporting the role of inflammation in fatigue in clinical populations, particularly from cancer and cancer-related fatigue research (11, 52). During cancer treatment, the increase in circulating concentrations of inflammatory markers, such as C-reactive protein (CRP) and interleukin (IL-6), was related to the development of an overall feeling of fatigue (53–55). Inflammation has also been associated with higher levels of post-cancer persistent fatigue. For example, breast cancer survivors who are fatigued, i.e., reporting lower levels of energy or vitality, show signs of activated inflammatory processes with increased concentrations of circulating inflammatory markers, as well as increased *ex vivo* inflammatory response to an immune challenge (56–58). In this population, higher levels of circulating CRP have shown a positive correlation with overall levels of fatigue even after adjusting for several confounders, such as obesity, self-rated health, depression, and insomnia symptoms (59). Although most of these studies used unidimensional assessments of overall fatigue, some have reported an association between inflammation and multidimensional fatigue, probing for different aspects, such as physical or mental fatigue, in cancer patients. These studies indicate that inflammation in cancer patients and survivors may affect particularly the physical rather than the mental aspects of fatigue (60–63). Further investigation, aiming specifically at assessing the role of inflammation in the different dimensions of fatigue, is needed to determine whether inflammation indeed leads mainly to the development of physical fatigue, or whether it also contributes to the cognitive/mental and motivational aspects of fatigue in cancer patients.

The extreme clinical form of fatigue in CFS/ME has been the subject of extensive study and provides a good model for assessing the potential role of inflammation in the development of fatigue (6). CFS/ME is a debilitating multisystem condition primarily defined by a disabling fatigue for more than 6 months, along with several other symptoms, including pain and cognitive changes (64). Due to the nature of the illness, a broad array of fatigue questionnaires are used for this group of patients, measuring several aspects of fatigue (65). One of the key symptoms is “postexertional fatigue” (64), which, interestingly, appears to be somewhat unique for this patient group (65). Although the underlying mechanisms of this disease are complex, a clear immunological component stands out; CFS/ME often appears following an infection, and some of the most promising treatments are immunomodulatory (64, 66). Furthermore, an extensive literature indicates that patients suffering from CFS/ME exhibit increased systemic production of pro-inflammatory cytokines [e.g., IL-6 or tumor necrosis factor (TNF)-α] and higher CRP at baseline as well as after immune stimulation, compared to non-fatigued individuals (67–72). Altered cytokine production is also associated with the intensity of fatigue symptoms in CFS/ME patients (73, 74). Regarding multidimensionality, CRP concentrations have been found to associate with physical health-related quality of life but not with mental health-related quality of life in a mixed sample of healthy individuals, individuals with high level of fatigue, and patients with CFS/ME (72). Although these measures of health-related quality of life do not specifically assess fatigue, this study highlights the fact that inflammation may be only related to certain dimensions of symptoms or symptom clusters. This has also been indicated in other clinical conditions, such as type 2 diabetes. Type 2 diabetes is characterized by low-grade but chronically increased concentrations of inflammatory markers, found to be closely associated with mental fatigue and lack of motivation, but not with physical fatigue (10). In other patient groups, inflammation has been found to relate to several dimensions of fatigue, both physical and mental. This is the case for patients suffering from MS, in which inflammation correlates with both physical and cognitive dimensions of fatigue, as well as with sleepiness (75).

Taken together, inflammation may be a key player in the development of pathological fatigue. However, the few studies assessing the role of inflammation in fatigue using a multidimensional perspective indicate that inflammation may not always relate to all dimensions of fatigue. In patient groups with long-term fatigue and comorbidity, other factors may thus be of greater importance for some aspects. Importantly, we do not advocate an “inflammation-specific type of fatigue,” but argue that the fatigue dimensions that are affected by inflammation may vary in different medical conditions. This is of high importance when considering the development of anti-inflammatory therapeutic interventions to improve fatigue in patients. Pharmacological treatments aiming at blocking the actions of cytokines, such as inhibitors of TNF-α, have been found to clinically reduce fatigue in patients suffering from rheumatoid arthritis or psoriasis (76, 77). However, if inflammation relates only to a specific aspect of fatigue in a certain population, the use of cytokine inhibitors may only improve certain types of fatigue. For instance, medication with a monoclonal antibody against IL-1β (XOMA052) was found to affect physical, but not cognitive fatigue in type 2 diabetes (78). A better understanding of the effect of inflammation on the multidimensional aspects of fatigue in medical conditions is therefore essential for long-term clinical applications.

### Inflammation and Fatigue in the General Population

Inflammation does not only relate to fatigue in clinical populations, but there is also a connection between inflammatory activity and fatigue in the healthy population. Inflammation, as measured with CRP levels, has been found to predict the development of fatigue in healthy subjects 5 years later, even after adjusting for several confounders (79). In addition, a recent study has shown that CRP concentrations in the general population are associated with higher fatigue and reduced sleep quality, but not altered mood or concentration difficulties (80). An earlier study using a multidimensional assessment of fatigue in healthy individuals, however, contradicts these findings, showing that depressive symptoms and adiposity were better predictors of overall and physical fatigue than inflammation (81). Similar results were found in older individuals, for which the association of circulating concentrations of CRP and IL-6 with overall and physical fatigue was no longer significant when adjusting for depressive symptoms or adiposity (82). These results may at least partially be due to sex differences, as the authors also reported a significant relationship, independently of depressive symptoms and adiposity, between CRP levels and fatigue intensity in women but not in men (83).

Inflammation thus seems to contribute to the development of fatigue even in the general population, but a multidimensional assessment is generally lacking. This is unfortunate given that it may prevent the understanding of the pathophysiology of fatigue. Indeed, the different dimensions of fatigue may involve distinct underlying neurological processes. One illustrating example relies on motivational changes, which may drive, at least partially, inflammation-induced fatigue (84). Decreased motivation results from specific alterations in reward-related neuronal processes, involving notably the mesolimbic dopamine pathway (85).

## Potential Neuronal Mechanisms Underlying Dimensions of Inflammatory-Induced Central Fatigue

As described in Box 1, during the activation of the immune system, the inflammatory cytokine signals reach the brain (38, 39), inducing changes in neurotransmitter and neuroendocrine systems, and leading to behavioral changes (7, 8). For example, cytokines can inhibit the synthesis of neurotransmitters, such as dopamine or serotonin, by activating specific enzymes involved in the rate-limiting steps of their biosynthesis (86, 87). These alterations in neurotransmitter systems ultimately lead to modifications in neuronal functions, which in turn induce behavioral changes collectively called *sickness behavior*. Sickness behavior includes fatigue, reduced activity, altered mood state, changes in cognitive functions, and reduced appetite. Sickness behavior is an adaptive process allowing the body to rest and to redirect energy toward fighting infections (17, 88). Although most of the effects of cytokines on the CNS have been demonstrated with high levels of circulating cytokines (e.g., after an immune challenge or during immunotherapy), evidence also suggests that low-grade levels are enough to affect the brain (89, 90). Interestingly, the specific modifications of CNS functions during immune system activation can help infer some mechanisms that likely underlie inflammation-induced central fatigue. Notably, imaging studies of immune challenges highlight changes in activation of the anterior cingulate cortex (ACC), the anterior insula, and the ventral striatum (87, 91). As these areas have also been associated with fatigue in several medical conditions, they seem likely to underlie inflammation-induced fatigue symptoms (92). Here, we take this one step further and propose that specific functional brain alterations induced by inflammation may contribute to the development of the different dimensions of fatigue.

### The Basal Ganglia

Given that motivation is a core feature of fatigue and that inflammation has been shown to modulate reward-related processes (88, 93, 94), it is possible that these reward-related processes are involved in the effect of inflammation on fatigue (84). The mesolimbic dopamine pathway, linking the ventral tegmental area to the nucleus accumbens (in the ventral striatum), is essential in the modulation of motivation (85, 95) and particularly in effort-related motivational behaviors (96). This “non-motor part” of the basal ganglia has been suggested as a critical mechanism for the development of central fatigue (13, 97). Altered dopamine processes in the ventral striatum can lead to an effort–reward imbalance, with increased perception of energy costs of actions and/or decreased expectation of reward or benefits (16). This can lead to the feeling of physical and/or mental fatigue, although the underlying issue is a reduced motivation to perform physical or cognitive tasks (98). Additionally, even though fatigue research has focused mainly on the motivation pathway of the basal ganglia, the motor pathway may be involved as well. Decreased volume and activation of the putamen, caudate, and pallidum have been described in fatigued patients with MS or CFS, and are associated with the intensity of fatigue symptoms (99, 100).

Several lines of research indicate that inflammation may induce the development of fatigue, specifically through reduced motivation *via* alterations in basal ganglia functions. Cytokines are known to affect dopamine function (see Box 1), which leads to modifications in basal ganglia activity, such as the mesolimbic dopamine pathway. A reduced activation of the ventral striatum in response to hedonic reward has indeed been observed after an immune challenge (93, 101). This functional change has been suggested to underlie the development of cytokine-induced fatigue (102, 103). Furthermore, fatigue, but also psychomotor slowing, that develops after the instauration of immunotherapy appears to relate to modifications in dopamine function (while mood and cognitive dimensions rather relate to serotonin function) (49–51, 104). After the commencement of cytokine therapy, patients also exhibit increased glucose metabolism in the basal ganglia, which is associated with symptoms of fatigue and reduced motivation as assessed with the MFI (105, 106). In addition, the reduction of ventral striatal activity in response to reward observed during immunotherapy is associated with reduced motivation, reduced activity, as well as depressive symptoms (101). Immunotherapy-induced physical fatigue, measured as decreased energy, was found to relate to increased basal activity both in the putamen and the ventral striatum (107), but motivational changes were not assessed in this study. Additionally, a recent study nicely illustrates the specific contribution of the ventral striatum in cytokine-induced fatigue (108). In this study, very early ventral striatal alterations induced by immunotherapy (4 h after the initiation of immunotherapy) significantly predicted the later development of fatigue (4 weeks follow-up). Importantly, these changes in striatal function did not predict mood symptoms, which supports the idea that inflammatory effects on the brain may be separated into distinct circuits that underlie the different parts of sickness behavior, some of which drive fatigue specifically. Nevertheless, fatigue was not assessed in a multidimensionality perspective in this study, and specific changes in motivation were not evaluated.

These studies highlight the potential contribution of the basal ganglia, particularly the mesolimbic pathway, in inflammation-induced fatigue. While these studies have been conducted in conditions of high-level activation of the immune system, some data on older adults suggest that inflammation at a low-grade state is sufficient to induce alterations in the dopamine system, contributing to the development of fatigue (41). However, although some of these studies have used multidimensional assessments of fatigue, including reduced motivation, those assessing the effect of inflammation on basal ganglia changes usually measure either fatigue or motivational changes, but rarely the two together.

### The ACC

The ACC has been implicated in inflammation-driven processes in several studies (105, 109, 110), and we suggest that this area could be related to the cognitive aspects of fatigue. The ACC, in particular the dorsal part, is involved in conflict monitoring (111–113) and in cognitive control (114). Activation of the dorsal ACC seems to signal the adjustment of cognitive processes according to the difficulty or cognitive demand of the task (115). Interestingly, it has been suggested that the feeling of cognitive/mental fatigue may arise from an increased cerebral effort to maintain a satisfactory performance (116). An increased activation of the ACC during a motor or mental task has been shown in fatigued patients with CFS or MS (117, 118) and was associated with a feeling of having to exert more effort (118). Thus, it is possible that the stronger activation of the ACC signals a need for increased cognitive processing, leading to a feeling of mental fatigue.

Inflammation-induced sickness may represent a more demanding mental state for an individual than full health does, as indicated by a decline in cognitive abilities during immune activation (119). Altered activation of the ACC, mostly an increase in the dorsal part, has been repeatedly reported during activation of the immune system (105, 109, 110, 120, 121). Importantly, the dorsal ACC was the structure most strongly activated during an attentional task in patients treated with immunotherapy, in comparison to control subjects (109). This activation also correlated with number of errors, in line with the involvement of the dorsal ACC in conflict monitoring. Furthermore, inflammation-induced fatigue during a more acute model (typhoid vaccination) was found to significantly correlate with the activation of the ACC during a mental conflicting task (the Stroop task) (122). This was, however, not the case for those feeling fatigued after placebo, suggesting a specific mechanism of inflammation on ACC functions in the development of fatigue.

### The Insula

There is a growing interest in the potential role of the insular cortex in inflammation-induced fatigue. This brain area is considered a main hub for the perception of the physiological condition of the body, so-called interoceptive signals, and it has been suggested as the central structure for “human awareness” (123, 124). Speculatively, a tiresome task would require insular involvement for the brain to interpret the associated bodily signals, and the behavioral output to restore homeostasis and promote rest would be the feeling of fatigue. An increased responsiveness of the insula to interoceptive signals would, therefore, make individuals more prone to feeling fatigued. Interestingly, it has repeatedly been shown that inflammation increases insular activity (121, 122, 125–127). Two studies even show a relationship between inflammation-induced insular function and fatigue development (122, 126). In addition, patients with MS, a condition characterized by both alterations of inflammatory processes and fatigue, exhibit increased activation of the insula during a motor task (128). To speculate further, inflammation may thus induce increased sensitivity to interoceptive signals, through stronger insula reactivity, leading to a more rapid development of an overall feeling of fatigue when performing tasks.

### Other Central Processes

It is not our intention to reduce fatigue processes to the three brain structures above, and additional brain structures could very well contribute to fatigue in the situation of immune activation. For instance, the self-regulatory and cognitive functions of the pre-frontal cortices are likely to play an important role in the modulation of fatigue (129, 130). Nevertheless, our aim was to highlight that different neuronal functions may underlie different dimensions of fatigue and that more (multidimensional) studies are needed to comprehend the involvement of inflammation in its pathogenesis.

In addition, beyond the functions of specific brain areas, changes in the connections between structures may also underlie the development of fatigue (131–133). This research is still in its infancy, but bears great potential for understanding potential mechanisms. Inflammation has been shown to affect intrinsic connectivity (134–136) and, for instance, the connectivity between the insula and mid-cingulate cortex seems associated with the inflammation-induced state of malaise and discomfort, in line with the interoceptive role of insula (136). Although this has not yet been studied in relation to fatigue, it is probable that altered connections between structures, in addition to specific structural changes, also contribute to the development of fatigue (97, 137).

**Figure 2:**
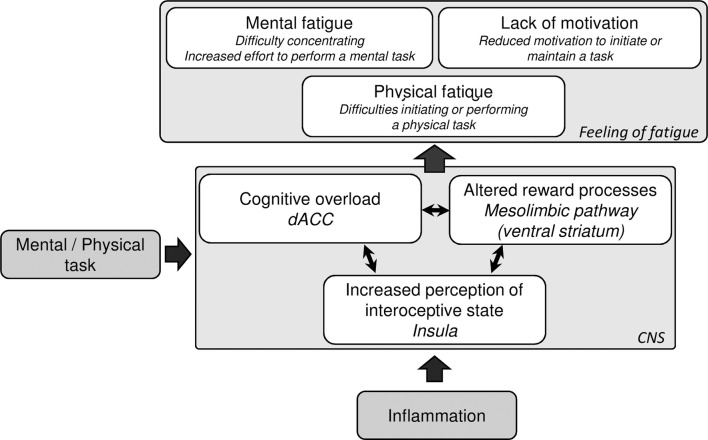
**Suggested mechanisms of inflammation-induced central fatigue**. Inflammation is known to modulate several neuronal processes, including dorsal anterior cingulate cortex function, the mesolimbic pathway, and insula reactivity. During a physical or mental task, the inflammation-induced altered activation of these neuronal processes may contribute to the feeling of fatigue.

In summary, inflammation appears to induce changes in neuronal functions that in turn may contribute to the development of fatigue (Figure 2). Although the specific involvement of the cerebral structures for the different dimensions of fatigue remains to be elucidated, we argue that a higher cognitive load during inflammation could lead to a feeling of mental fatigue and depend on changes in ACC function. The mesolimbic reward system on the other hand, may be involved in the dimension of fatigue that relates to lack of motivation, a feature that may be particularly prominent in inflammation-induced fatigue. Finally, higher sensitivity to interoceptive signals may induce an overall feeling of fatigue. While inflammation may be involved in all these processes, it is also possible that, in some medical conditions or patient subpopulations, inflammation contributes to only one or some of these processes. It is therefore important to characterize the specific dimensions of fatigue that develop in patient populations, and assess the role of inflammation. For instance, some aspects of fatigue may be derived from sleep alterations or changes in hormonal regulation in clinical populations, e.g., of insulin or cortisol, which can modulate brain functions including those in the above-mentioned areas (138–140).

## Concluding Remarks and Future Considerations

In this review, we have highlighted the potential role of inflammation in the development of pathological central fatigue. Importantly, we wanted to illustrate the need for multidimensional assessments of fatigue when assessing the role of inflammation, given that fatigue contains distinct features that may be explained by separate central mechanisms and may be specific to different medical conditions. Studying fatigue using a multidimensional perspective also appears highly relevant for the development of therapeutic interventions that target inflammation in order to improve fatigue. In cases where inflammation contributes to only some aspects of fatigue, the use of anti-inflammatory therapies may not be sufficient to improve the feeling of fatigue.

It is therefore important to disentangle the dimensions of fatigue if we are to understand the pathophysiological role of inflammation in this symptom. While the use of single, general measures of fatigue is sometimes preferable, depending on the researcher’s or clinician’s need (27), the choice of the fatigue measurement(s) should be carefully considered with regard to the study aims (141). This is especially true since no gold-standard measure exists at this point. However, some recommendations can be made for when the aim is to understand the underlying pathophysiological processes. Several self-report scales of multidimensional fatigue are available, such as the MFI (14), the Swedish Occupational Fatigue Inventory (SOFI) (21), the Checklist Individual Strength (CIS) (142), and the Multidimensional Fatigue Symptoms Inventory (MFSI) (143). It is also important to take into account the time span of fatigue. Hence, while one may assess the feeling of fatigue over a long period of time when referring to pathological fatigue (e.g., 1 or 2 weeks as assessed with the MFI, CIS, or MFSI), measuring acute changes in fatigue when assessing the effects of inflammation is also crucial. This can be done by repeated assessments of the level of fatigue that the subject feels at the time of scale completion, as measured with the SOFI (which, however, lacks a mental fatigue dimension) or visual analog scales, such as the Visual Analogue Scale for Fatigue (VAS-F) (144), which only focuses on physical fatigue. The use of an acute measurement of fatigue also allows for evaluating subjective fatigue induced by physical, mental, or motivational tasks. Using a fatigue-induced task can help define fatigue in a more precise way than when relying solely on reports that pertain to the past few weeks. Needless to say, the development of new scales would be beneficial to the field. These should include both chronic and acute fatigue, as well as the multidimensional features. In the meantime, we suggest using a combination of different scales. In addition, self-report scales could be combined with objective measures of fatigue, such as reduction of performance during a physical or mental task. Effort-related reward tasks, such as the Effort Expenditure for Reward Task (EEfRT) (145), in which subjects receive a monetary reward for effort, may also help in understanding the role of motivational changes in fatigue (146). Nevertheless, it is preferable to combine these objective assessments of fatigue with subjective measures, given that objective fatigue is not always associated with subjective reports of fatigue (28), and that fatigue is first and foremost a subjective experience.

In conclusion, although fatigue is increasingly taken into account by clinicians, the study of this symptom remains limited by being restricted to overall fatigue, which, as highlighted in this review, may encompass many different mechanisms. While inflammation may be involved in the development of fatigue, the specific underlying mechanisms remain poorly understood, perhaps partly because the different dimensions of fatigue are too rarely explored. The mechanisms underlying other inflammation-induced neuropsychiatric symptoms have been inferred thanks to multidimensional assessments (51), and this strategy should be pursued when studying inflammation-induced fatigue as well. Fatigue is a critical and highly disabling symptom for many patient groups and individuals. We argue for the need of adequate multidimensional assessments in order to increase the understanding of the mechanisms underlying inflammation-induced fatigue, as well as for the development of effective therapeutic interventions.

## Author Contributions

JL, BK, and TS have written and approved the final version of this manuscript.

## Conflict of Interest Statement

The authors declare that the research was conducted in the absence of any commercial or financial relationships that could be construed as a potential conflict of interest. The reviewer AF-H declared a shared affiliation, though no other collaboration, with the authors to the handling Editor, who ensured that the process nevertheless met the standards of a fair and objective review.
